# Race, ethnicity, and gender representation in clinical case vignettes: a 20-year comparison between two institutions

**DOI:** 10.1186/s12909-022-03665-4

**Published:** 2022-07-30

**Authors:** Courtney R. Lee, Kurt O. Gilliland, Gary L. Beck Dallaghan, Sue Tolleson-Rinehart

**Affiliations:** 1 University of Pennsylvania Perelman School of Medicine, Philadelphia, PA USA; 2 UNC School of Medicine, 108 Taylor Hall, CB 7321, NC 27599 Chapel Hill, USA

**Keywords:** Cultural awareness, Social determinants of health, Medical student, Clinical case discussions, Race, Gender

## Abstract

The medical case vignette has long been used in medical student education and frequently includes demographic variables such as race, ethnicity and gender. However, inclusion of demographic variables without context may reinforce assumptions and biases. Yet, the absence of race, sexual orientation, and social determinants of health may reinforce a hidden curriculum that reflects cultural blindness. This replication study compared proportions of race, ethnicity, and gender with University of Minnesota (UMN) findings. This study sought to determine if there has been progress in the representation of demographic characteristics in case vignettes. Methods: University of North Carolina (UNC) case vignettes from 2015–2016 were analyzed and compared to UMN case vignettes from 1996–1998. Data included mentions of race, ethnicity, gender and social determinants of health. Results: In the 278 UNC vignettes, white race was noted in 19.7% of cases, black race was in 7.9% cases, and 76.6% of cases were unspecified. In the 983 UMN vignettes, white race was recorded in 2.85% cases, and black race in 0.41% cases. The institutions were significantly different in the proportion of their cases depicting race (0.20; 95% CI (0.15, 0.25)). Males were represented in the majority of vignettes. Discussion: Comparing case vignettes results from two medical schools suggests that reporting explicit demographic diversity was not significantly different. The findings illustrate that sex was the demographic characteristic consistently described, where males were over-represented. Based on these findings, greater cultural diversity as it intersects with social determinants of health is needed in medical student education.

## Background

The medical case vignette is a long-standing communication tool for health professionals. In medical training, case histories provide students with learning topics used in individual and group problem-based solving. Given the importance of problem-based learning in pre-clinical training, the case vignette plays a key role in introducing students to clinical practice and presents the first opportunity for students to ‘meet’ patients [1, 2]. As a result, the structure, language, and perspective of case vignettes are at the precise intersection between the explicit messages of medical practice and the implicit assumptions of medical culture [3–5]. Case vignettes help shape the values and attitudes of students’ professional development.

Case histories possess a well-defined structure typically including demographic variables such as race, ethnicity and gender. Medical providers commonly use case histories to communicate. However, recent literature has investigated how this approach to case histories can be problematic to student education. Tsai et al. demonstrated that race was often mentioned within case vignettes in association with biological or epidemiologic risk factors that potentially reinforced racial biases [6]. This study, however, examined less than six months of the first year- and second year-curricula within one institution. In a review of the United States Medical Licensing Examination Step 1 question bank, Ripp and Braun found that of the 455 cases that mentioned race and/or ethnicity, 412 cases (90.5%) mentioned race without clear relevance to the answer, question stem, or educational goal [7]. While robust data, this study does not address longitudinal exposure to race and ethnicity in case vignettes. Including race and ethnicity within case vignettes can have negative consequences especially when presented in a manner that makes it unclear how or why race and ethnicity were included in the history at all. Conversely, does the absence of race and ethnicity have the potential to reinforce biases as well?

To understand how depictions of race and ethnicity within case histories can signal implicit messages about diversity and inclusion within the larger medical community, the cultural competence continuum was applied as the conceptual framework [8]. Cultural competence can be defined as an organization’s or individual’s ability to understand, communicate, and interact effectively across cultures [9–11]. The cultural competence continuum includes six categories: cultural destructiveness, cultural incapacity, cultural blindness, cultural pre-competence, cultural competence, and cultural proficiency.

While organizations can present along any aspect of the continuum, this study concentrated on the role of cultural blindness. Two important underpinnings of cultural blindness are the notions of “universal applicability” and “assumptions of normativity,” which accept the concept that patients’ cultures do not meaningfully influence care. The increasing awareness of racial disparities and the importance of providing patient-centered care, has ignited an ongoing dilemma within medical education. Krishnan et al. recently described a guide to revising cases to improve the representation of race and culture [11]. Nevertheless, the question remains whether cases should portray race or ethnicity to students, and, if so, then how. Can case histories effectively incorporate race and ethnicity into medical education, without reinforcing biases and stereotypes [12, 13]? Are the consequences of incorporating race and ethnicity without context as detrimental as not including them at all? The issue is a complicated and nuanced one, but we submit that the absence of representation across race, sexual orientation, and social determinants of health (SDH) may send implicit messages that reinforce cultural blindness [14–17].

This study aims to identify the proportions of race and ethnicity represented in case vignettes at two institutions. By replicating and comparing results reported 20 years ago, this study sought to identify if there has been any significant change in the representation of race and ethnicity in case history discussions.

## Materials and methods

Case vignettes from the University of North Carolina School of Medicine (UNC) were reviewed. Demographics present in 278 small group case vignette catalog available during academic year (AY) 2015–2016 for first-year and second-year students were collected. Case vignettes used during UNC small group discussions were chosen due to ease of extraction from the learning management system. Our pre-clinical curriculum spans three semesters, where case vignettes are consistently used from year to year. In accordance with the UNC Institutional Review Board, this study was exempt from review since it involves review of curricular materials.

The primary data collected included mentions of race and ethnicity in case vignettes. The secondary data points included the mention of gender, sexual orientation, SDH and health behaviors that were specified in cases. These variables were reported as percent of total cases.

A case vignette was defined as (1) an individual illustration with at least two sociodemographic descriptors (e.g., age and sex, or race and age), and (2) an illustration of a normal or pathological process or disease. The following sociodemographic variables were coded: race, ethnicity, gender, gender identity, and sexual orientation. The definition of social determinants of health was based on the World Health Organization definition, which describes social determinants of health as the conditions in which people are born, grow, live, work, and age. These circumstances are shaped by distribution of money, power, and resources at global, national, and local levels [18, 19].

The United States Department of Health and Human Services, in *Healthy People 2020*, categorized SDH into five areas: economic stability, education, social and community context, health and health care, neighborhood and built environment [20]. To evaluate underlying variables in each area, occupation was coded as an indicator of economic stability, insurance status as a proxy of health care and access, housing as an indicator of neighborhood and environment, and educational attainment as the proxy of education. Given the limitations of this study social and community context were unable to be assessed. Other health demographics included: tobacco use, alcohol use, substance use, physical activity, and body mass index. The chief complaint, course, and organ system for each vignette were also included in the data set.

One author (CL) was responsible for coding each case and a second author (STR) selected random cases to verify the coding. To avoid making assumptions about the case authors’ intentions, we used the term “unspecified” (rather than “absent” or “missing”) to describe information not explicitly mentioned within the case’s text. Since the number of cases representing SDH and health behavior were limited in both the UNC and UMN data, both sets of vignettes were collapsed into “specified” versus “unspecified.”

We gathered results from a University of Minnesota (UMN) study conducted by Sandra Turbes, Erin Krebs, and Sara Axtell, who examined cases from small groups, didactics, and examinations between the academic years of 1996 and 1998 for first-year and second-year students [21]. The UMN information we use here was obtained from their study results and not from our own primary data collection or analysis. We included the UMN results in our study because theirs was one of the first studies, to our knowledge, to investigate such characterization of cases.

United States Census Bureau data was used as a benchmark when comparing the proportion of race in case histories related to each state’s current population. State population data from 2000 census data most accurately reflected the population when the UMN data were collected [22]. Similarly, 2010 census data best approximated state population data for the UNC cases [23]. This data allowed us to make appropriate proportional comparisons of demographic data.

To determine the existence of a statistically significant difference between the institutions’ expression of demographics, the percentage of the presentation of demographics at either institution was calculated using difference of proportions tests. Stata 14 (StataCorp, LLP, College Station, TX) was used for comparisons.

## Results

From UNC, 278 vignettes were reviewed to compare to results of 983 vignettes from UMN.

In the UNC vignettes, white race was noted in 19.7% of cases, black race was noted in 7.9% vignettes, and 76.6% of vignettes were unspecified. Based on census data obtained from 2010 and multi-year estimates for 2018, in North Carolina 70.6% of the population was white, 22.2% were black, 1.6% were American Indian, and 3.2% were Native Hawaiian [23]. In the UMN data, white race was recorded in 2.85% cases, and black race was coded in 0.41% cases, in a state in which, in the 2000 census, 89.4% of people were white, 3.5% were black, 1.1% were American Indian and Alaska Native, 2.9% were Asian [23]. The institutions were significantly different in the proportion of their vignettes that depicted race (0.20; 95% CI (0.15, 0.25)). At both institutions, “black” and “white” were the predominant racial descriptors (Table 1).

**Table 1:** Demographic characteristics featured in case histories from North Carolina and Minnesota

Demographic Characteristics OF CASES	North Carolina N (%)	Minnesota N (%)	Difference in proportions (95% CI)
Sex			
Male	146 (52.3)	526 (53.5)	-0.01 (-0.08, 0.06)
Female	127 (45.7)	416 (42.3)	
Unspecified	5 (1.8)	29 (3.0)	
Intersex		12 (1.2)	
Gender			
Unspecified	278 (100.0)	983 (100.0)	-
Cisgender			
Transgender			
Sexual Orientation			
Unspecified	276 (99.3)	977 (99.4)	-0.001 (-0.012, 0.001)
Heterosexual	2 (0.7)	3 (0.31)	
Homosexual		2 (0.20)	
Other		1 (0.10)	
Race			
Unspecified	213 (76.6)	951 (96.7)	0.20 (0.15, 0.25)
White	42 (19.7)	28 (2.9)	
Black	22 (7.9)	4 (0.4)	
Other	1 (0.4)		
Region of Origin/Ethnicity			
Unspecified	240 (86.3)	970 (98.7)	0.12 (0.08, 0.17)
Europe	3 (1.1)	5 (0.51)	
South and Central America			
Caribbean	1 (0.36)	1 (0.10)	
Middle East			
Africa	2 (0.72)	3 (31)	
Asia	1 (0.36)	2 (0.20)	
North America	31 (11.2)	1 (0.10)	

SOURCES: The table presents data collected at UNC compared to the study conducted at UMN [22]

Both UMN and UNC identified patient sex in the overwhelming majority of vignettes (98.2% at UNC and 95.8% at UMN; Table 1); a small majority of cases were male at both institutions (52.3% at UNC; 53.2% at UMN;(-0.01; 95% CI (-0.08, 0.06)).

Neither school presented any vignettes explicitly involving transgender patients. However, UMN included 12 intersex cases (1.2%). Sexual orientation was unspecified in nearly all of the vignettes; neither UNC nor UMN differ in this regard (-0.001; 95% CI (-0.012, 0.001)).

Behavioral characteristics and SDH data were only obtained from UNC, because the UMN study did not report this information. Behavioral characteristics such as tobacco, alcohol, and substance use were specified occasionally in vignettes at UNC, but physical activity and body mass index (BMI) were usually unspecified (Table 2). Variables associated with SDH such as education level, housing status, and insurance status were rarely described in vignettes (Table 3).

**Table 2:** Percentage of behavioral characteristic featured in case histories

Behavioral characteristics and Social Determinants of Health (SDH)	{UNIVERSITY} SOM N (%)
Tobacco Use	
Unspecified	224 (80.6)
Yes	32 (11.5)
No	38 (15.7)
Alcohol Use	
Unspecified	240 (86.3)
Yes	20 (7.2)
No	18 (6.5)
Substance Use	
Unspecified	259 (93.2)
Yes	4 (1.4)
No	15 (5.4)
Body Mass Index (BMI)	
Unspecified	259 (93.2)
Physical Activity	
Unspecified	262 (94.2)

Results from the case history analysis at UNC

**Table 3:** Percentage of case histories at North Carolina without Social determinants of health described

Education Level	
Unspecified	243 (93.2)
At least some high school/GED	11 (4.0)
Some college or greater	24 (8.6)
Occupation	
Unspecified	242 (87.1)
Housing	
Unspecified	258 (93.2)
Insurance Status	
Unspecified	277 (99.6)

Results from the case history analysis at UNC

## Discussion

The evidence from the analysis of our vignettes indicated reporting race and ethnicity is not distinctly different for a study conducted 20 years ago. We assessed the apparent pattern of the case vignettes by applying the cultural competence continuum as a conceptual framework.

Universal applicability implies that medicine is scientifically based and “impartial” and, thus, one can assume it is not influenced by sociopolitical or cultural events [24]. Universal applicability also suggests normativity, which assumes the dominant culture is the reference group to which everything is compared [12, 13]. The assumption prescribes characteristics of the norm to the dominant culture in all situations. We postulate that the lack of representation may reinforce the assumption of the dominant racial group unless otherwise specified, which has resulted in medical schools actively addressing social justice issues within the curriculum.

For both UNC and UMN, the findings illustrate that sex was the only demographic characteristic consistently described in at least half of the case vignettes. Even then, at both schools, males were over-represented, rather than proportionately presented. This finding may be rooted in several reasons. Gender was likely represented most consistently because it has traditionally been used when considering the prevalence, incidence, or risk factors for diseases. Additionally, gender can influence management or diagnostic work up for certain diseases. This may also explain why gender representation did not substantially vary within the last 20 years. The disproportionate representation of males may reflect differences in prevalence between males and females across diseases albeit not always consistently. Turbes et al., for example, demonstrated that the percentage of female case vignettes underrepresented the prevalence of hypertension, myocardial infarction, and heart failure amongst women [21]. Medical educators may consider how disproportionate representation of biological sex can send implicit messages and consider using an objective benchmark when determining the proportion of gender within case vignettes.

The absence of demographic diversity may convey to students that elements of culture are not relevant to health care delivery unless under circumstances where culture is salient to risk assessment or diagnosis; these are the problems of universal applicability and assumptions of normativity we introduced. There is evidence to illustrate that cultural indifference biases provider behaviors. One study offered evidence that race and sex resulted in differential recommendations for cardiac catheterization [25]. Other studies have found a link between stereotyping and anesthesiologists’ decisions about pain management [26, 27]. Racial differences in hospital admissions for pneumonia and congestive heart failure have also been identified [28]. For this reason, it is important to not only address what is explicitly taught about diversity within case histories, but what is implicitly taught as well.

Tobacco and alcohol use were commonly featured examples of behavioral characteristics in vignettes, most likely because substance use is associated with several common diseases, such as lung cancer and heart failure [29, 30]. SDH were virtually absent from the case vignettes at UNC. The absence of SDH information may stem from concerns that these details may over-complicate a case for students early in their training. Additionally, clinical teachers may view SDH as secondary to the biomedical curriculum or may feel unprepared to discuss the nuances of the SDH. When they begin to care for patients, students will be faced with navigating the challenging relationship between behavior, SDH, and health outcomes. Avoiding the nuances created by SDH early in training may prevent students from discussing these fundamental features of patients’ lives early in training.

This view can streamline biomedical learning but cannot improve medical students’ ability to deliver care across cultures [31, 32]. Using the cultural competence continuum as a conceptual framework, we can begin to synthesize how patterns in the explicit curriculum can convey implicit messaging that influences students’ attitudes and behaviors about diversity, equity and inclusion [33]. With a better understanding of these underlying assumptions and how they persist over the course of multiple years, we can think more critically about how to address them and further investigate their role on student development.

This study was limited by the inability to control for potential regional and cultural differences between the two schools, as well as for the effects of time. Additionally, there may be unmeasured confounders not assessed, which may have influenced our results. The cultural climate of a school may contribute to the inclusion or exclusion of variables in case vignettes. Given the disparities and racial biases uncovered by the coronavirus-19 pandemic and police brutality, these findings further demonstrate the need for medical schools to appropriately represent race and ethnicity in their curricula using a conceptual framework such as the cultural competence continuum [34]. Another limitation is that the Turbes et al. case vignettes at UMN were drawn from lectures, exams, and small groups, whereas the UNC case vignettes exclusively came from small groups [21]. Since case vignettes may include varying levels of information based on the setting in which they are presented, it is possible that including case vignettes from UNC lectures and exams may have improved representation in our findings.

## Conclusion

From habitual exposure to case histories, students learn the necessary information for disease management and diagnosis. Patterns in the sociocultural characteristics featured in vignettes can convey messages that potentially undermine efforts to move medical education toward cultural competence. While including race and ethnicity may reinforce stereotypes and exacerbate biases, excluding sociodemographic variables to the extent we have found in this study may suggest to students that information about a patient’s sociocultural experience is either unimportant or assumed. This is of particular significance since future physicians will interact with diverse patients and be expected to provide culturally competent care. Identifying implicit messages expressed to medical students allows medical schools and clinical faculty to address those messages and promote inclusive behavior and attitudes. The cultural competence continuum offers a framework to assess implicit aspects of course materials and assessments in a way that brings cultural diversity of case examples into the mainstream. We must stop using race and ethnicity in ways that imply a deviation from the norm and focus on how to include all individuals in case vignettes. Whether based on prevalence data or the cultural competence continuum, involving more diverse members of society conveys to students that diversity is important to health care delivery, particularly in light of the inequalities brought to the forefront in 2020. Future steps include investigating how case vignettes impact students’ attitudes about race and ethnicity and whether this varies when case histories better reflect society. With the insights from these findings, educators can identify areas to incorporate cultural diversity into case vignettes so that medical students may experience the nuances of the cultural competent care at the earliest point of their education.

## Data Availability

Data is available upon request from the lead author, Dr. Courtney Lee.
